# Pancreatic cancer cells require an EGF receptor-mediated autocrine pathway for proliferation in serum-free conditions

**DOI:** 10.1054/bjoc.2001.1698

**Published:** 2001-04

**Authors:** L O Murphy, M W Cluck, S Lovas, F Ötvös, R F Murphy, A V Schally, J Permert, J Larsson, J A Knezetic, T E Adrian

**Affiliations:** Department of Biomedical Sciences, Creighton University School of Medicine, Omaha, NE, 68178; Department of Surgery, Karolinska Institute at Huddinge Hospital, Huddinge, Sweden; Endocrine, Polypeptide and Cancer Institute, VA Medical Center and Tulane University School of Medicine, New Orleans, LA, USA

**Keywords:** autocrine, EGF-receptor, MAPK AP-1

## Abstract

In-vitro and in-vivo studies have shown that autocrine growth factors and receptors are frequently expressed in human malignancies. Few of these studies, however, provide evidence that the identified autocrine pathway is functional. In this study, a functional autocrine growth pathway in pancreatic cancer has been identified using an in-vitro cell culture system. When pancreatic cancer cells were grown without change of medium, proliferation was greater than when either medium was replaced frequently (HPAF, CAPAN-2, PANC-1 or SW1990) or cells were grown in the presence of the EGF receptor tyrosine kinase inhibitor AG1478 or the MEK inhibitor PD098059 (HPAF or CAPAN-2). Activity of extracellular-regulated kinases (ERK) 1 and 2 and c- *jun* and c- *fos* mRNA levels were significantly elevated in CAPAN-2 cells cultured continuously in serum-free medium. Collectively, the observations indicate that the EGF receptor and the ERK MAP kinase pathway mediate autocrine signals. In contrast to previous reports, the GRP and IGF-I receptors were shown not to be required for autocrine effects on pancreatic cancer cell proliferation. Autocrine stimulation of the EGF receptor can contribute to sustained mitogenic activity and proliferation of pancreatic cancer cells. © 2001 Cancer Research Campaign http://www.bjcancer.com


*British Journal of Cancer* (2001) **84**(7), 926–935
© 2001 Cancer Research Campaign
doi: 10.1054/ bjoc.2000.1698, available online at http://www.idealibrary.com on IDEAL®

http://www.bjcancer.com

### Pancreatic cancer cells require an EGF receptor-mediated autocrine pathway for proliferation in serum-free conditions

**LO Murphy[1], MW Cluck[1], S Lovas[1], F Ötvös[1], RF Murphy[1], AV Schally,[2] J Permert[3], J Larsson[3], JA Knezetic[1] and TE Adrian[1]**

[1]Department of Biomedical Sciences, Creighton University School of Medicine, Omaha, NE, 68178; [2]Department of Surgery, Karolinska Institute at Huddinge Hospital, Huddinge, Sweden; [3]Endocrine, Polypeptide and Cancer Institute, VA Medical Center and Tulane University School of Medicine, New Orleans, LA, USA

**Summary** In-vitro and in-vivo studies have shown that autocrine growth factors and receptors are frequently expressed in human malignancies. Few of these studies, however, provide evidence that the identified autocrine pathway is functional. In this study, a functional autocrine growth pathway in pancreatic cancer has been identified using an in-vitro cell culture system. When pancreatic cancer cells were grown without change of medium, proliferation was greater than when either medium was replaced frequently (HPAF, CAPAN-2, PANC-1 or SW1990) or cells were grown in the presence of the EGF receptor tyrosine kinase inhibitor AG1478 or the MEK inhibitor PD098059 (HPAF or CAPAN-2). Activity of extracellular-regulated kinases (ERK) 1 and 2 and c-*jun* and c-*fos* mRNA levels were significantly elevated in CAPAN-2 cells cultured continuously in serum-free medium. Collectively, the observations indicate that the EGF receptor and the ERK MAP kinase pathway mediate autocrine signals. In contrast to previous reports, the GRP and IGF-I receptors were shown not to be required for autocrine effects on pancreatic cancer cell proliferation. Autocrine stimulation of the EGF receptor can contribute to sustained mitogenic activity and proliferation of pancreatic cancer cells. © 2001 Cancer Research Campaign http://www.bjcancer.com

**Keywords**: autocrine; EGF-receptor; MAPK AP-1

Human pancreatic carcinoma is an extremely aggressive disease that currently is the fourth leading cause of cancer death in males and females in the USA (Landis et al, 1999). Several studies have shown that genes such as *p53*, *Ki-ras*, *p16* and *DPC4* are frequently mutated in this cancer (Apple et al, 1996; Hahn et al, 1996; Lynch et al, 1996). These genetic aberrations are similar to those detected in other human cancers and are unlikely to be the reason why pancreatic cancer is more aggressive than others. It is possible that activation of mitogenic pathways by autocrine growth factors contributes to the devastating nature of pancreatic cancer (Korc, 1991).

Autocrine growth can occur when a cell co-expresses a membrane-bound growth factor receptor and soluble ligand(s) for this receptor. Over the past decade, a variety of autocrine growth pathways have been implicated in the pathogenesis of human cancers. Techniques such as radioligand binding analysis, column chromatography and immunohistochemistry have demonstrated the presence of receptors and ligands in tumour tissues and in cell lines. In pancreatic cancer cells, these receptors and ligands are thought to collectively provide the cell with a trophic signal (Korc, 1991; Wang et al, 1996). Previous studies have demonstrated that exposure of these cells to recombinant growth factors such as transforming growth factor α (TGFα) (Korc, 1991), amphiregulin (Munehiro et al, 1994) or insulin-like growth factor-1 (IGF-I) (Ohmura et al, 1990) produces modest effects on cell proliferation. It is possible that stimulation of growth pathways by endogenous growth factors, produced by cells in culture, could explain the poor response to exogenous factors. In the current study, we have investigated the relative importance of different autocrine growth pathways in pancreatic cancer. The results presented below indicate that growth factor receptors and ligands expressed by pancreatic cancer cells do not necessarily constitute a functional autocrine growth pathway in vitro.

*Received 11 May 2000*
*Revised 19 December 2000*
*Accepted 21 December 2000*

*Correspondence to:* TE Adrian

### MATERIALS AND METHODS

### Materials

Porcine heparin was obtained from Sigma Chemical Company (St. Louis, MO). Recombinant human EGF, TGFα and amphiregulin (long form) were from Intergen (Purchase, NY). The EGF receptor tyrosine kinase inhibitor AG1478 and the MEK inhibitor PD098059 were obtained from Calbiochem (La Jolla, CA) and dissolved in dimethyl sulphoxide. The final concentration of solvent in the well was not more than 0.1% (shown to have no effect on proliferation). The TGFα-neutralizing polyclonal antiserum was purchased from R&D Systems (Minneapolis, MN). The amphiregulin-neutralizing monoclonal antibody, 16.21.28, was provided by Dr Bruce Cohen, Bristol Myers Squibb (Seattle, WA) and the IGF-I receptor monoclonal antibody, αIR3 (Kull et al, 1983), was provided by Dr. Steve Jacobs (Burroughs Wellcome Co., Research Triangle Park, NC). The GRP receptor antagonist RC-3950-II and the synthetic human EGF B-loop analogue, $[Abu^{20,31}]hEGF(20–31)\text{-}NH_2$, were synthesized and purified as previously described (Ötvös et al, 1994; Qin et al, 1995). All reagents used to immunoprecipitate and assay ERK1 and ERK2 activity were supplied by New England Biolabs Incorporated (Beverly, MA) and used as described in the kit instruction manual (version 1.3). The Prism Ready Reaction DyeDeoxy Terminator Cycle Sequencing Kit was purchased from Perkin Elmer (Foster City, CA). *Ampliscribe* T7 RNA polymerase solution, nucleotide triphosphates and dithiothreitol were obtained from Epicenter Technologies (Madison, WI). RT-PCR reagents were purchased from Perkin Elmer (Foster City, CA).

### Receptor binding

Analysis was performed as described previously (Wang et al, 1996). Cells were harvested, resuspended in binding buffer and seeded in 96-well filter plates (100 000 cells per well). [$^{125}$I]-labelled EGF, GRP, IGF-I or insulin (20 000 cpm) was added to each well along with various concentrations of unlabelled EGF, bombesin or insulin. Filter plates were incubated at 37˚C in an atmosphere of 5% $CO_2$ for 120 minutes. Binding was terminated by rapid filtration using a 96-well plate vacuum manifold. The individual wells were subsequently washed twice with 250 µl of ice-cold wash buffer and the cell-surface-associated radioactivity was counted.

### Gel chromatography of TGFα, bombesin and IGF-I-like immunoreactivity in conditioned medium

The peptide fraction of HPAF conditioned medium (1 L) was adsorbed on a SepPak cartridge and eluted with a solution (2 ml) of 30% acetonitrile in water containing 0.1% trifluoroacetic acid. This solution (100 µl) was applied to a Superdex Peptide HR10/30 size exclusion column (0.5 ml min$^{-1}$, 0.6 Mpa) which had been equilibrated with the same solvent. Recombinant human TGFα, synthetic mammalian bombesin analogues and recombinant IGF-I were similarly chromatographed. Peptide-like immunoreactivity in column fractions was measured using specific radioimmunoassays. The TGFα and IGF-I assays show no significant cross-reactivity with other growth factors (Skullman et al, 1994). The bombesin assay measures gastrin-releasing peptide (GRP) and neuromedin C (NMC) equally (Wang et al, 1996).

### Cell culture

HPAF, CAPAN-2, SW1990 and PANC-1 cells were obtained from the American Type Culture Collection (Rockville, MD). The HPAF cells used in this study had been adapted to serum-free culture and were routinely maintained in IMDM. McCoy's 5A Medium (CAPAN-2), L-15 Medium Leibovitz (SW1990), or DMEM (PANC-1) media were supplemented with 10% fetal bovine serum. All cell culture media contained 1% antibiotic-antimycotic solution and all the cell lines were cultured at 37˚C in a humidified sterile atmosphere of 5% $CO_2$ except SW1990, which was grown in a $CO_2$-free environment. Each series of experiments was performed on cells within 5 passages.

### Growth studies

HPAF, PANC-1, SW1990 (35 000 well$^{-1}$) or CAPAN-2 cells (50 000 well$^{-1}$) were seeded in 24-well plates and grown for 72 hours in the above culture conditions. Media were then removed and the cells were washed with phosphate buffer (500 µl). The appropriate serum-free medium (2 ml) was then added to each well. In control experiments, cells continued to receive fresh serum-free medium every 12 hours (Figure 1). Otherwise, cells remained in the same medium for the duration of the experiment (continuous culture). Inhibitors used to implicate specific receptors or ligands in the autocrine process were added to medium at the beginning of the continuous culture. For cell cycle analysis, ERK1 and ERK2 assays and c-*fos* and c-*jun* mRNA quantification, CAPAN-2 cells (1.5 million) were cultured in T-25 culture flasks for different times and processed as described below.

### [$^3$H-*methyl*] thymidine incorporation

At the end of incubations, medium was removed and cells were washed with phosphate buffer. Cells were incubated with 500 µl of [$^3$H-*methyl*] thymidine solution (0.5 mCi well$^{-1}$) for 2 hours at 37˚C. After the labelling period, the cells were precipitated with glacial acetic acid:methanol (1:3, v/v) and then washed with a solution of 80% (v/v) methanol in water. DNA in the acid-insoluble fraction was dissolved by addition of 0.5 M NaOH (300 µl) and radioactivity contained in aliquots (100 µl) was counted in a liquid scintillation counter (LKB Wallac, Turku, Finland).

### Cell number

The number of cells in each well was determined by harvesting the cells with trypsin-EDTA solution (500 µl well$^{-1}$) and counting cells in an aliquot (100 µl) on a Z1 Coulter Counter (Coulter Scientific Instruments, Hialeah, FL).

### Cell cycle analysis

Cells were cultured as described above and harvested with trypsin-EDTA solution (2 ml) which was then diluted with phosphate buffer (5 ml). Samples were centrifuged at 1000 ***g*** for 10 minutes and pellets resuspended in phosphate buffer (500 µl). Ice-cold ethanol (1 ml) was added to each cell suspension, which was then incubated for 12 hours at 4˚C. Tube contents were centrifuged at 500 ***g*** for 10 minutes and the resultant pellets were washed with phosphate buffer (1 ml). Following centrifugation at 500 ***g*** for 10 minutes, pellets were reconstituted in the Telford reagent (1 ml) and shaken for 12 hours in the dark at 4˚C. Fluorescence emission between 580 nm and 750 nm was determined using a FACScan.

### Immunoprecipitation and Western blot analysis

Cells were washed with ice-cold phosphate buffer (5 ml), incubated with lysis buffer (500 µl) on ice for 1 min and snap-frozen on liquid nitrogen. The thawed samples were collected using a cell scraper and sonicated 3 times on ice. Lysates were clarified by centrifugation at 12 000 ***g*** for 10 minutes at 4˚C and protein concentration in the samples was determined using the BioRad DC protein kit. ERK1 and ERK2 were immunoprecipitated from lysates and assayed for activity according to the manufacturer.

### Quantitative RT-PCR

The phosphormidite method of olignonucleotide synthesis was used to synthesize primers for RT-PCR of c-*fos* (forward:

*© 2001 Cancer Research Campaign* 

5′-ACCAGTCCGGACCTGCAGTGG-3′; reverse: 5′-GCGGCATT-TGGCTGCAGCCATC-3) or c-*jun* (forward: 5′-CTGTCCCCCAT-CGACATGGAGT-3′;reverse:5′–AAGGAGTACTACAGAAGC-ATCTAC-3′) mRNA. Competitor RNA molecules for c-*fos* or c-*jun* were produced by PCR-amplifying sequences from the pGL2 basic plasmid (c-*fos*; 1470–1588, c-*jun*; 1063–1619) and cloning them into pCR2.1. The recognition sequences for the primers listed above were also engineered into these sequences. Clones were sequenced to verify correct insert orientation and then transcribed in vitro using T7 RNA polymerase (0.1 μg DNA per incubation). The transcription template (pCR2.1) was eliminated with DNase I (verified by PCR analysis) and RNA remaining was precipitated using 2.5 M ammonium acetate. Competitor RNA was first quantified by measuring the absorbance at 260 nm and then stored at –80˚C. Quantitative analysis of c-*fos* or c-*jun* mRNAs was performed by incubating a sample of total cellular RNA (0.5 μg) with various amounts of competitor RNA as indicated in the legend to Figure 7. The mRNA and competitor RNA were co-reverse transcribed and amplified using either the c-*fos* or c-*jun* primer pairs.

### Data analysis

Statistical analysis of data was performed using a paired analysis of variance with Dunnett's multiple comparisons test as a post-test for individual comparisons to appropriate control data. Digital analysis of GST-Elk-1 phosphorylation and quantitative RT-PCR reaction products was performed using the UV Gel Documentation System. For the later, the $\log_{10}$ of the cRNA/mRNA band intensity was plotted against the $\log_{10}$ of the cRNA concentration of each reaction tube. Linear regression analysis of the data points was performed using the Prism II software package. The inverse $\log_{10}$ of the x-intercept corresponded to the amount of mRNA in the total RNA analysed and mRNA levels were expressed as moles per μg of total RNA.

### RESULTS

### HPAF cells express components for different autocrine growth pathways

The binding of [$^{125}$I]-EGF, -GRP or IGF-I to HPAF cells was specific and could be competitively prevented with unlabelled ligand (Figure 1A–C). Approximately 100-fold more unlabelled insulin was required to inhibit the binding of [$^{125}$I]-IGF-I than [$^{125}$I]-insulin to HPAF cells (Figure 1C). The chromatographs for gel permeation analysis of the peptide fractions from HPAF-conditioned medium showed that TGFα, GRP and IGF-I-like immunoreactivity were eluted in the same position as the corresponding synthetic peptide (Figure 1D–F). GRP-like immunoreactivity was detected in a major peak corresponding to GRP and in a minor immunoreactive peak with the $V_e$ of neuromedin C (Figure 1E).

### Frequent change of medium prevents autocrine growth stimulation

The [$^3$H] thymidine incorporation into HPAF cells after 72, 96 and 120 hours of continuous culture was significantly higher than into cells receiving fresh serum-free medium every 12 hours (Figure 2). Cell number also increased significantly after 96 and 120 hours but not after 72 hours (Figure 2). [$^3$H-*methyl*] thymidine incorporation into HPAF cells, receiving fresh serum-free medium every 12 hours, during a 120-hour time course, did not significantly change (72 h, 4580 ± 480 cpm; 96 h, 4660 ± 780 cpm; 120 h, 5330 ± 620 cpm). Frequent changes of medium also had a significant inhibitory effect on the proliferation of CAPAN-2, SW1990 and PANC-1 pancreatic cancer cells ($P < 0.01$, $n = 6$).

### EGF receptor function is required for autocrine growth factor action in pancreatic cancer cells

The increase in [$^3$H] thymidine incorporation into HPAF DNA due to soluble endogenous growth factors was completely inhibited by the EGF receptor tyrosine kinase inhibitor, AG1478 or an EGF analogue, [Abu$^{20,31}$]hEGF(20–31)-$NH_2$ (Figure 3A). Both AG1478 and [Abu$^{20,31}$]hEGF(20–31)-$NH_2$ inhibited thymidine incorporation in a concentration-dependent manner (Figure 4A and C, respectively). In contrast, the GRP receptor antagonist, RC3950-II, or the IGF-I receptor blocking antibody, αIR3, had no effect on the thymidine incorporation into cells cultured continuously for 4 days (Figure 3A and B). The [$^3$H] thymidine incorporation into HPAF cells cultured in the presence of 10 μM AG1478 was lower than that into cells receiving fresh serum-free medium every 12 hours. AG1478 also significantly reduced the number of HPAF cells grown continuously for 96 hours ($P < 0.05$, $n = 5$; data not shown). When HPAF cells were exposed to 1 nM EGF after 72 hours of continuous culture in serum-free medium, [$^3$H] thymidine incorporation increased significantly (Figure 4B and D). This increase was completely prevented by 10 μM AG1478 (Figure 4B) or [Abu$^{20,31}$]EGF(20–31)-$NH_2$ (Figure 4D).

Affinity-purified anti-TGFα IgG had no significant effect on the [$^3$H] thymidine incorporation into HPAF cells cultured continuously (Figure 5A) but did inhibit incorporation induced by exogenous TGFα (Figure 5B). In contrast, when cells were cultured continuously in the presence of heparin, [$^3$H] thymidine incorporation was inhibited in a concentration-dependent manner (Figure 5C). Heparin had no effect on EGF-induced [$^3$H] thymidine incorporation into HPAF cells but did inhibit the growth effect observed with recombinant amphiregulin (data not shown). The amphiregulin monoclonal antibody, 16.21.28, significantly inhibited the incorporation of [$^3$H] thymidine into these cells (Figure 5D).

### Autocrine activation of EGF receptors leads to ERK1/ERK2 activation

The cell permeable inhibitor of MEK1/2, PD098059, significantly inhibited the proliferation of HPAF cells (Figure 6A). The proliferation of CAPAN-2 cells cultured continuously in serum-free medium was completely inhibited by PD098059, as well as to AG1478 (Figure 6B). To detect changes in ERK-MAPK activation in CAPAN-2 cells, culture conditions were scaled up. Although exogenous EGF had no significant effect on the activity of ERK1/ERK2 in CAPAN-2 cells, PD098059 significantly inhibited the activity of ERK1/ERK2 in CAPAN-2 cells cultured continuously in serum-free medium. Using conditions identical to those for Figure 6C, the proportion of CAPAN-2 cells in S phase after 6 hours of continuous culture in serum-free medium

© 2001 Cancer Research Campaign was inhibited by AG1478 or PD098059 (Figure 6D).

### Autocrine activation of EGF receptors increases expression of AP-1 genes

The steady-state level of c-*fos* and c-*jun* mRNA in CAPAN-2 cells cultured continuously in serum-free medium increased transiently and after 6 hours, the mRNA levels returned to those measured in cells at the start (Figure 7A and B). When cells were cultured in the presence of AG1478 or PD098059, the level of *c-jun* mRNA

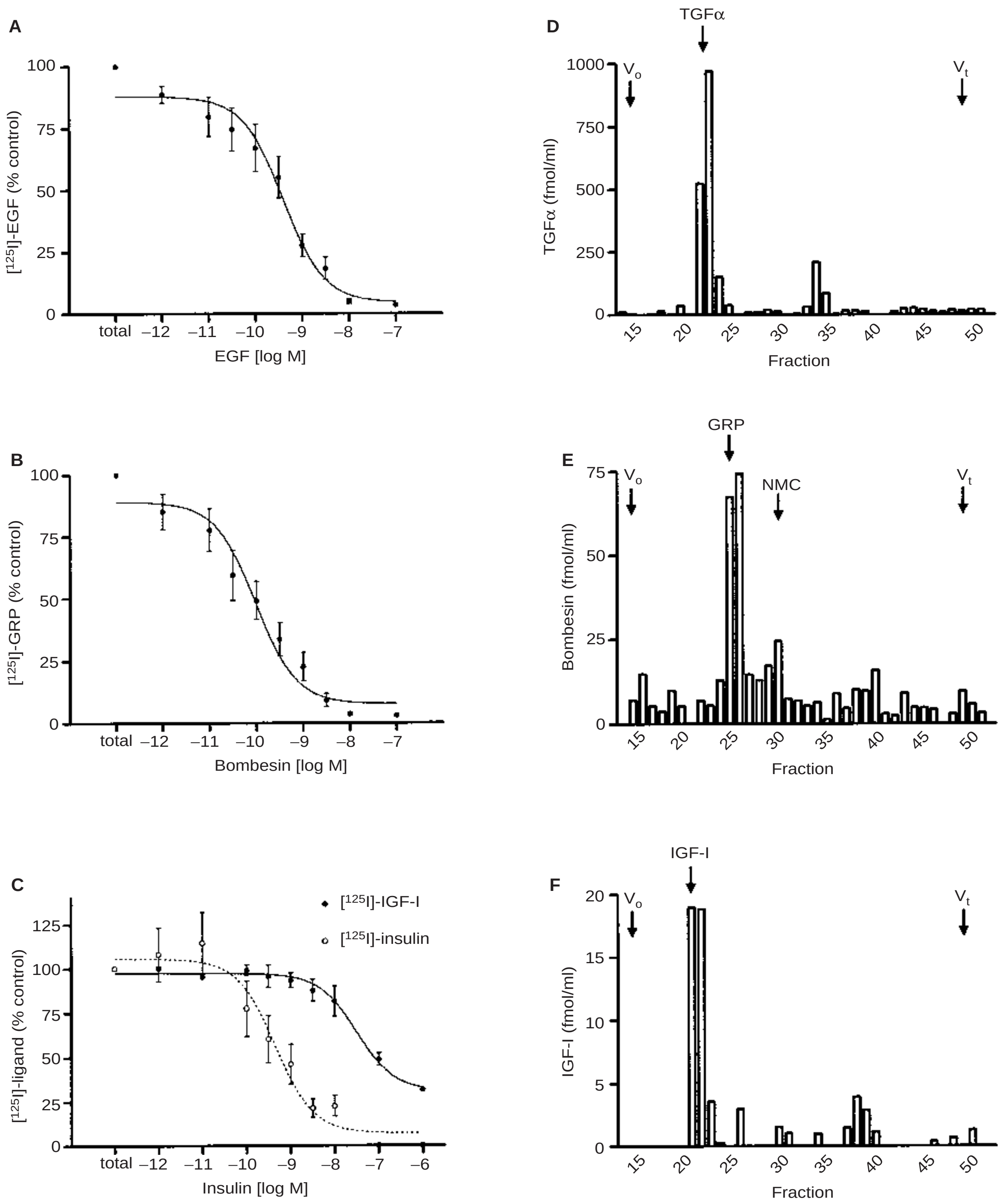

**Figure 1** HPAF cells express the components for an EGF, GRP or IGF-I autocrine growth pathway. Competitive binding analysis (**A–C**) was performed as described in the Methods. In panel **C**, the binding of [$^{125}$I]-IGF-I (solid symbols) or [$^{125}$I]-insulin (open symbols) to HPAF cells was competed with unlabelled insulin. Gel permeation of HPAF conditioned medium (**D–F**) was performed as described in the Methods. The void ($V_o$) and total volumes ($V_t$) of the Superdex Peptide column are indicated. The positions where recombinant TGFα, GRP, NMC or IGF-I elute from the column are indicated

*© 2001 Cancer Research Campaign*

was reduced to baseline levels (Figure 7C). The levels of c-*fos* mRNA after one hour of culture was similarly reduced by PD098059 or AG1478 (data not shown). FACs analysis showed the distribution of cells in S phase increased with time of culture in serum-free medium, peaking after 9 hours (data not shown). [$^3$H] thymidine incorporation similarly increased with time, reaching a maximum at 12 hours and decreasing thereafter (Figure 7D). In contrast, when cells were cultured in a larger volume of medium, 20 ml rather than 4 ml, the peak of [$^3$H] thymidine incorporation was sustained for a longer period (20–36 hours).

### DISCUSSION

In this study, the role of different autocrine growth factor pathways in the proliferation of human pancreatic cancer cells was examined. The expected observation that HPAF cells express binding sites for EGF and GRP is in agreement with the results of previous studies using other cell lines (Korc, 1991; Wang et al, 1996). Since unlabelled insulin was approximately 100-fold more effective in competitive inhibition of the binding of [$^{125}$I]-insulin compared to [$^{125}$I]-IGF-1, it is likely that HPAF cells express high-affinity binding sites for IGF-I. Soluble ligands for the EGF, GRP and IGF-I receptors were also present in HPAF-conditioned medium. These observations suggest that several autocrine growth pathways actively promote the proliferation of HPAF cells in vitro. When pancreatic cancer cells received frequent changes of medium, [$^3$H-*methyl*] thymidine incorporation and cell numbers were decreased, indicating that ligands such as TGFα, GRP or IGF-I potentially mediate autocrine stimulation of cells. Since [$^3$H-*methyl*] thymidine incorporation did not change in cells that received frequent medium changes, it seems likely that this approach is successful for making pancreatic cancer cells quiescent in vitro. Thus, the cell culture system employed here revealed a direct role for autocrine growth factors in the proliferation of pancreatic cancer cells.

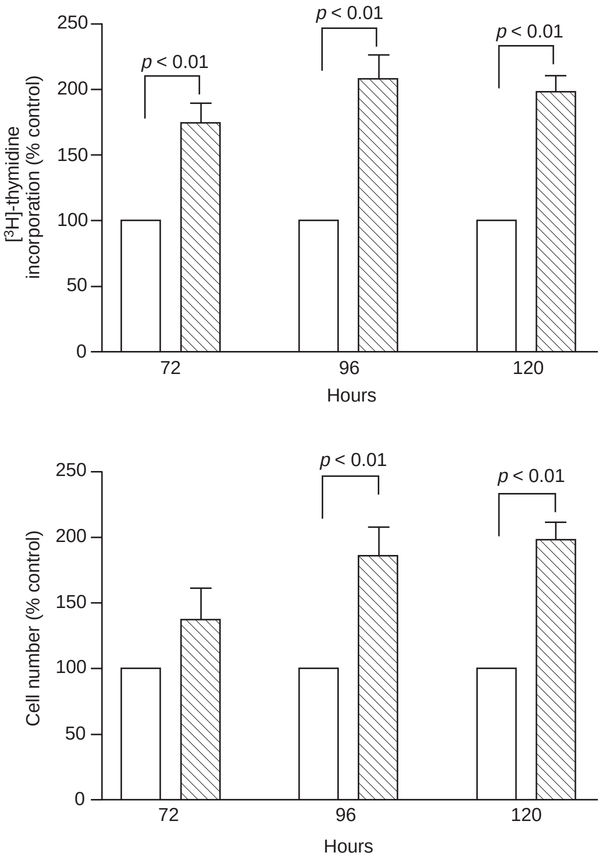

**Figure 2** The time course of [$^3$H-*methyl*] thymidine incorporation into HPAF DNA (upper panel) or the number of HPAF cells cultured in serum-free medium (lower panel). HPAF cells were either cultured in serum-free medium that was replaced every 12 hours (open bars) or continuously cultured (hatched bars). [$^3$H-*methyl*] thymidine incorporation into cells was determined as described in the Methods and is expressed as a percentage of the incorporation into cells receiving new medium every 12 hours. Data shown represents the mean ± SEM from 6 (thymidine incorporation) or 5 (cell number) separate experiments

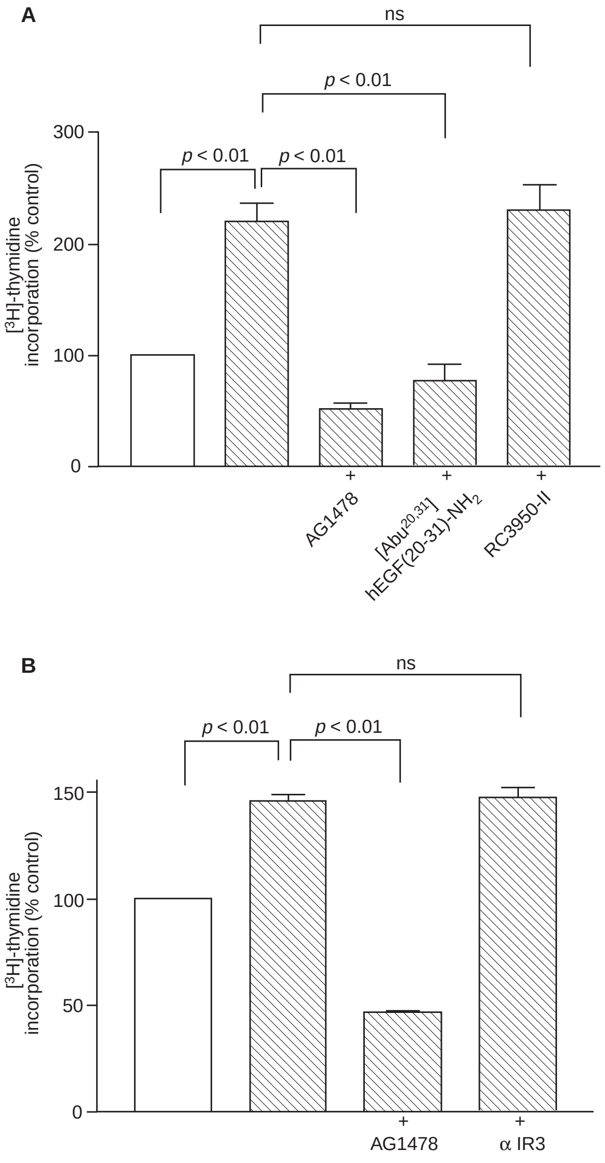

**Figure 3** Inhibitors of the EGF growth pathway block HPAF proliferation in serum-free medium. Cells were either cultured in medium which was replaced every 12 hours (open bars) throughout the experiment or continuously cultured (hatched bars) for 96 hours in the presence and absence of 10 μM AG1478, 300 μM [$Abu^{20,31}$]hEGF (20–31)-$NH_2$ or 1 μM RC3950-II. In (**B**), cells cultured continuously were treated with 10 μM AG1478 or 1 μg/ml αIR3. Data shown represents the mean ± SEM from 6 (**A**) or 3 (**B**) separate experiments

 *© 2001 Cancer Research Campaign*

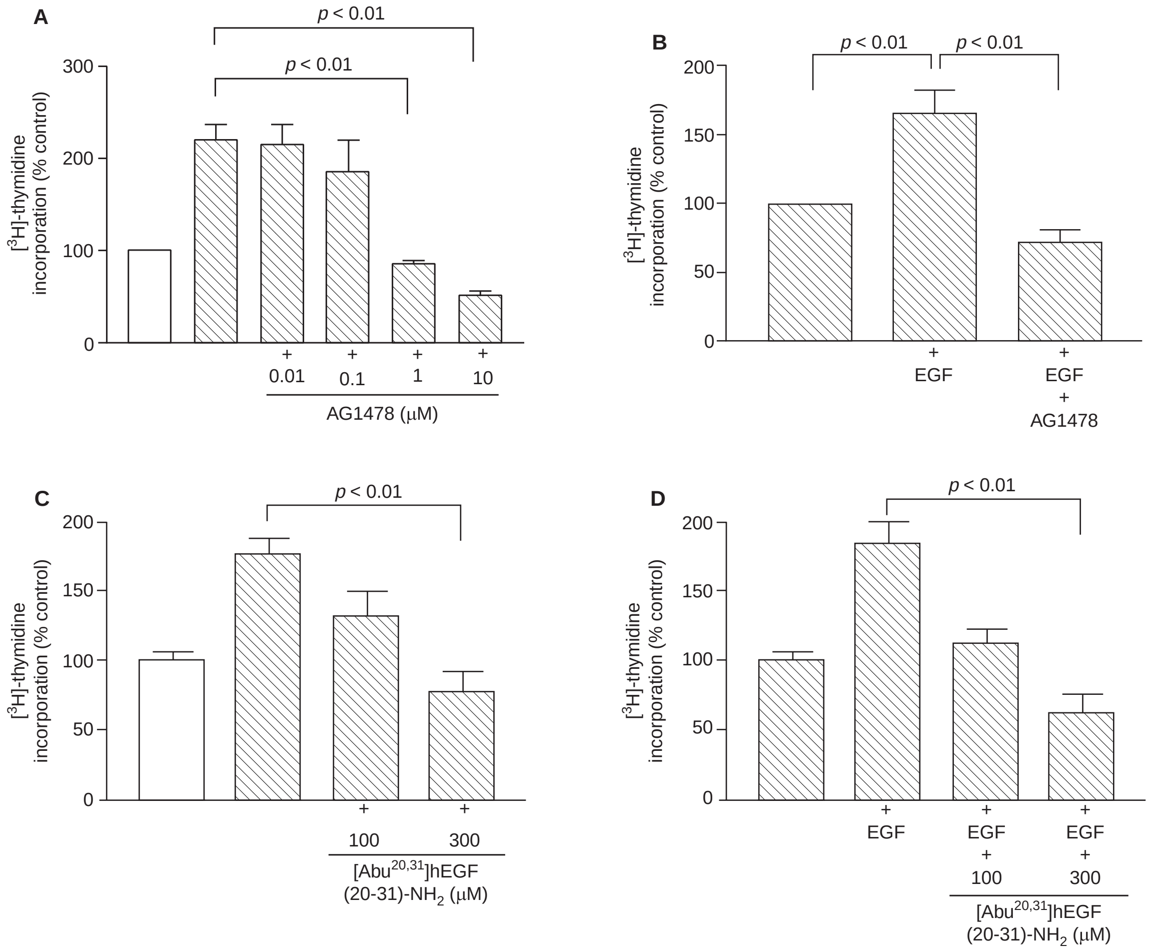

**Figure 4** The effect of AG1478 and of [Abu$^{20,31}$]hEGF (20–31)-$NH_2$ on [$^3$H-*methyl*] thymidine incorporation into HPAF cells cultured in serum-free medium. Cells were either cultured in medium which was replaced every 12 hours (open bars) throughout the experiment or continuously cultured (hatched bars) for 96 hours in the presence and absence of the indicated concentrations of AG1478 or [Abu$^{20,31}$]hEGF (20–31)-$NH_2$ (**A** and **C**). During the final 24 hours of continuous culture, cells were incubated with or without 1 nM EGF in the presence and absence of 10 μM AG1478 (**B**) or the indicated concentrations of [Abu$^{20,31}$]hEGF (20–31)-$NH_2$ (**D**). The data are expressed as a percentage of the proliferation of cells cultured in new medium every 12 hours (**A** and **C**) or continuously (**B** and **D**). Data shown represent the mean ± SEM from 5 separate experiments

Surprisingly, inhibiting the function of the EGF-receptor with an EGF receptor tyrosine kinase inhibitor, AG1478 (Osherov and Levitzki, 1994) but not a GRP receptor antagonist (RC3950-II) or an IGF-I receptor blocking antibody (αIR3) inhibited the proliferation of HPAF cells cultured continuously in serum free medium. AG1478 had no effect on IGF-1-stimulated proliferation in HPAF or PANC-1 cells; a further indication of the specificity of this agent for the EGF receptor tyrosine kinase (unpublished observations). CAPAN-2 and SW1990 pancreatic cancer cells were also sensitive to EGF receptor inhibition (unpublished observations). This indicates that the GRP and IGF-I receptor-mediated autocrine pathway is not functionally active under standard in-vitro conditions. We and others have suggested a role for a bombesin-like autocrine growth pathway in pancreatic cancer (Qin et al, 1994; Wang et al, 1996). These studies showed that the components of a bombesin-like autocrine growth pathway were expressed by pancreatic cancer cells and that a GRP receptor antagonist could inhibit the modest growth effect of exogenous GRP receptor ligands. The present study indicates that endogenous GRP and neuromedin C do not contribute to autocrine growth in pancreatic cancer cell lines.

The effect of [Abu$^{20,31}$]hEGF (20–31)-$NH_2$ on [$^3$H-*methyl*] thymidine incorporation provides further evidence that soluble endogenous EGF-like growth factors mediate autocrine growth effects in HPAF cells. This synthetic EGF analogue, competes for $^{125}$I-EGF binding in a concentration-dependent manner (unpublished observations). Like AG1478, [Abu$^{20,31}$] hEGF (20–31)-$NH_2$ reduced [$^3$H-*methyl*] thymidine incorporation below control levels, suggesting that the autocrine pathway detected may still be partly active in cells which received serum-free media every 12 hours (due to residual factors remaining after the medium change). Recently, potato carboxypeptidase inhibitor was shown to be an effective inhibitor of binding of EGF to receptors on pancreatic cancer cells but it had no effect on basal cell proliferation (Blanco-Aparicio et al, 1998). It is likely that the particular choice of cell density and medium volume in this study is responsible for the lack of effect.

Exogenous EGF was able to stimulate [$^3$H-*methyl*] thymidine incorporation into cells cultured continuously, suggesting that endogenous growth factors are not maximally stimulating cells during continuous culture. It is also possible that the signaling pathways activated by recombinant EGF are different from those

*© 2001 Cancer Research Campaign* 

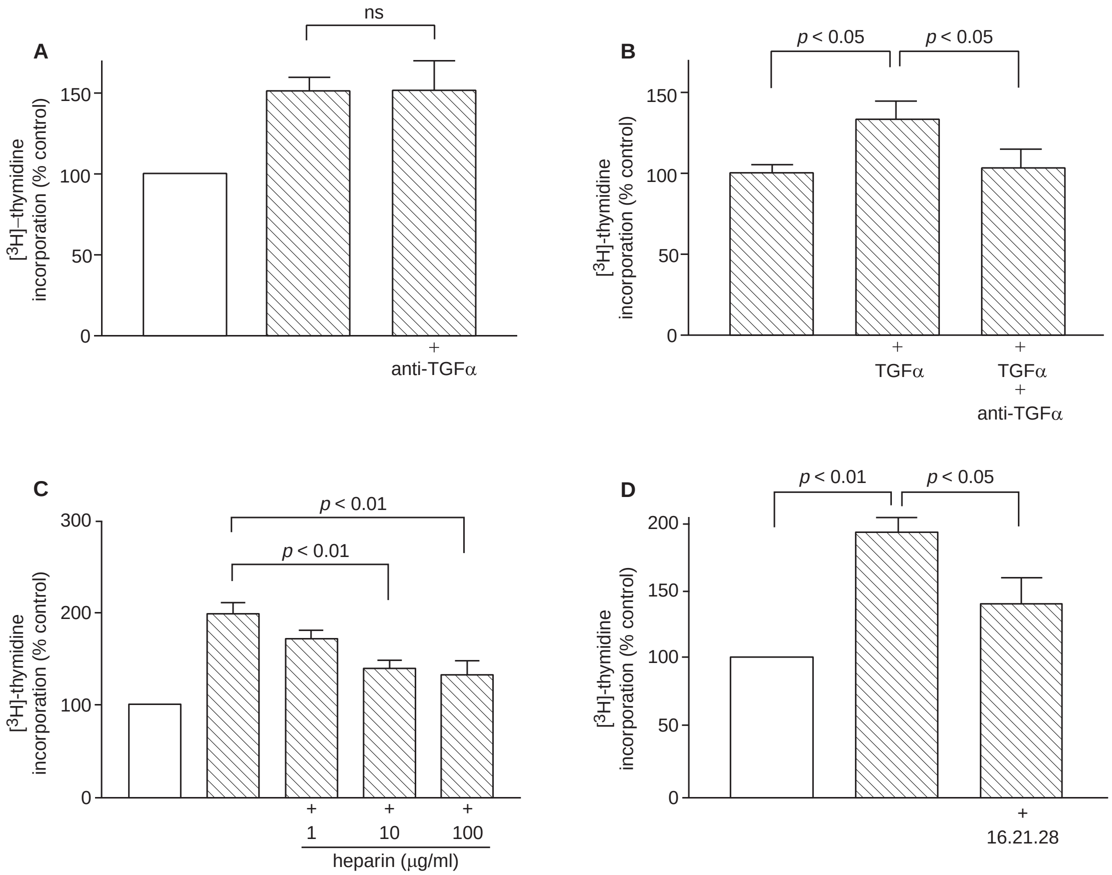

**Figure 5** Effect of anti-TGFα IgG, heparin and of 16.21.28 on the incorporation of [$^3$H-*methyl*] thymidine into HPAF cells. Cells either received fresh medium every 12 hours (open bars) or were cultured continuously (hatched) in the presence or absence of anti-TGFα IgG (anti-TGFα, 5 μg ml$^{-1}$) (**A**), heparin (**C**) or 16.21.28 (10 μg ml$^{-1}$) (**D**) for 96 hours. During the final 24 hours of continuous culture, cells were incubated with or without 1 nM TGFα in the presence and absence of anti-TGFα IgG (B). The data are expressed as a percentage of the [$^3$H-*methyl*] thymidine incorporation into cells cultured in new medium every 12 hours (**A**, **C** and **D**) or continuously (**B**). Data shown represent the mean ± SEM from 5 (**A** and **B**), 6 (**C**) or 3 (**D**) separate experiments activated by the endogenous EGF-like autocrine factors, leading to different effects on cell proliferation.

The inability of the anti-TGFα IgG to affect proliferation of HPAF or CAPAN-2 cells cultured continuously indicates that TGFα is not a functional autocrine growth factor, despite being readily detected in medium conditioned by pancreatic cancer cells. A previous study showed that an anti-TGFα IgG could partially inhibit PANC-1 cell proliferation (Wagner et al, 1996). It is likely that differences between pancreatic cancer cell lines and experimental procedures could explain this discrepancy. Nevertheless, in the present study, the effect of the anti-TGFα IgG on exogenous recombinant TGFα shows that the IgG can inhibit the bioactivity of a concentration of TGFα which exceeds that produced by pancreatic cancer cells under the experimental conditions used. It is also possible that the concentration of endogenous TGFα secreted into the culture medium may be too low to have a mitogenic effect. Other investigators have found that immuno-neutralizing endogenous TGFα does not inhibit cancer cell proliferation (Mulder and Brattain, 1989), but a TGFα antisense oligonucleotide does (Howell et al, 1998). This group and others (Hollande et al, 1997) have suggested that ligand-receptor complexes could be involved in signalling in the cytosol by an intracrine mechanism.

The inhibitory effect of heparin on the proliferation of HPAF cells cultured continuously in serum-free medium indicates that the functional autocrine growth factors produced by these cells contain heparin-binding domains. However, the proliferation of cells in the presence of heparin (100 μg ml$^{-1}$) remained higher than that of cells cultured in fresh medium every 12 hours which suggests that other EGF-like factors, in addition to those which bind heparin, are involved in autocrine growth regulation. Heparin has been used in the present study as a potential modulator of heparin-binding growth factor activity. Any change in cell proliferation in the presence of heparin can indicate the importance of heparin-binding growth factors (Cook et al, 1991; Jayson and Gallagher, 1997). The effect of heparin on EGF-stimulated proliferation of pancreatic cancer cells is consistent with another study which showed that heparin sulphate does not affect the TGFα-stimulated clonal growth of human keratinocytes (Cook et al, 1991). Other investigators have provided evidence to suggest that HB-EGF-like growth factor and amphiregulin may play important roles in growth regulation of human pancreatic cancer (Ebert et al, 1994; Schuger et al, 1996; Kobrin et al, 1994; Funatomi et al, 1997). Since heparin had no effect on EGF-stimulated proliferation in the present study, it is likely that exogenous heparin interacts with the basic amino acid residues in the amino-terminal regions of endogenous amphiregulin and/or HB-EGF (Schuger et al, 1996) thus preventing productive interactions between these

*© 2001 Cancer Research Campaign*

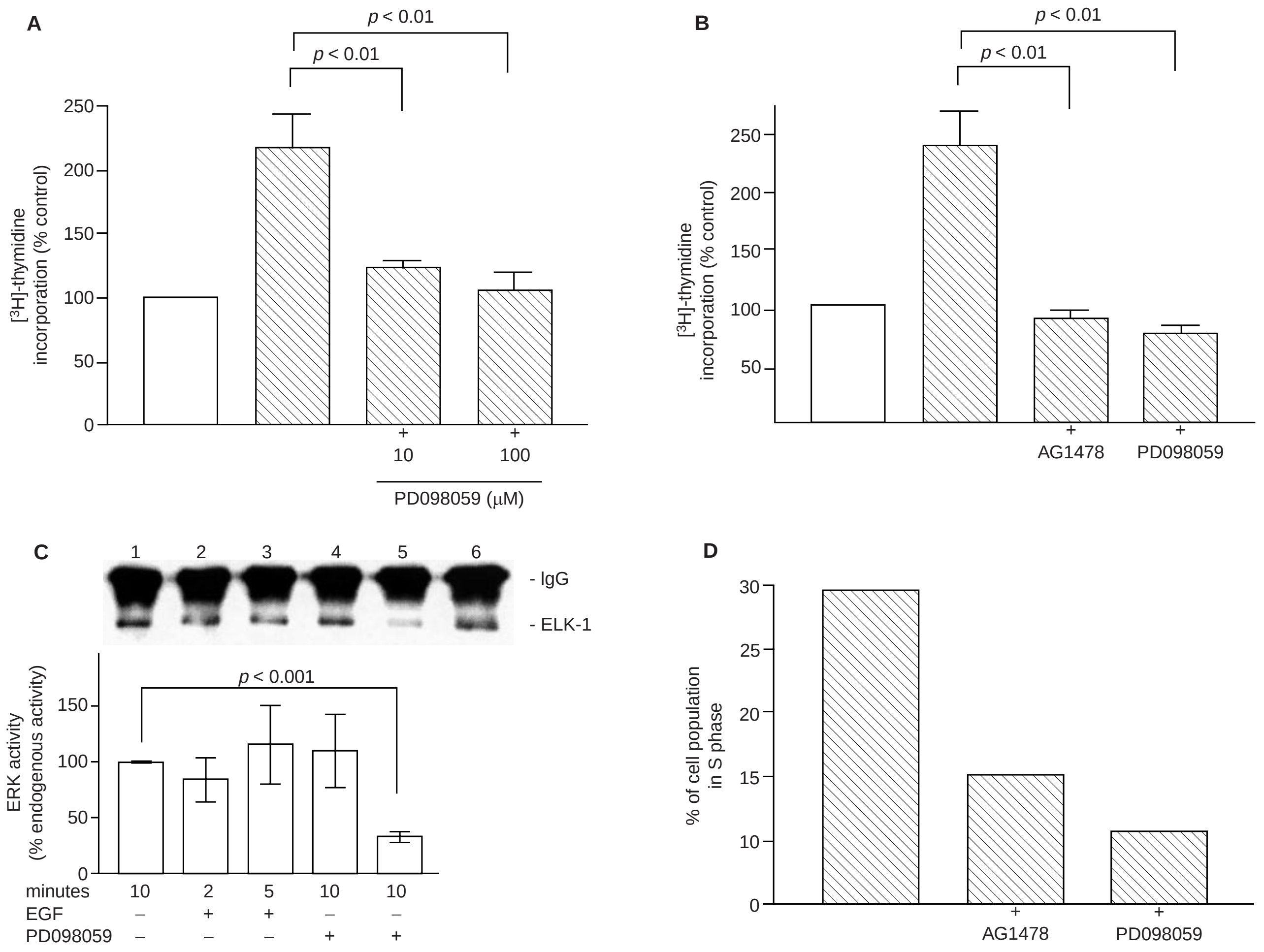

**Figure 6** Proliferation of pancreatic cancer cells in serum-free medium is sensitive to ERK-MAPK inhibition. HPAF cells (**A**) were cultured continuously for 96 hours with fresh media every 12 hours (open bars) or continuously (hatched bars) in the presence or absence of the indicated concentrations of PD098059. [$^3$H-*methyl*] thymidine incorporation was then assayed as described above. CAPAN-2 pancreatic cancer cells (**B**) were cultured as in (**A**) in the presence or absence of AG1478 (10 μM) or PD098059 (20 μM). Measurement of ERK1/ERK2 activity in CAPAN-2 cells (**C**) was performed as described in the Methods. CAPAN-2 cells were incubated with 1 nM EGF for 2, 5 or 10 minutes (lanes 2–4) or in serum-free medium in the presence (lane 5) or absence (lane 1) of 20 μM PD098059 for 10 minutes. GST-Elk-1 phosphorylation was quantified using a Gel Documentation System and was expressed as a percentage of the phosphorylation detected in the absence of EGF (endogenous activity, lane 1). In lane 6, the phosphorylation of GST-Elk-1 by recombinant active ERK2 (2 ng) is shown. A representative autoradiograph and the data from 3 individual experiments (mean ± SEM) is shown. The percentage of CAPAN-2 cells in S-phase after 6 hours of continuous culture in the presence or absence of AG1478 or PD098059 (**D**) was determined as described in the text ligands and the EGF receptor. Experiments using monoclonal antibody 16.21.28 confirms that HPAF cells proliferate in serum-free medium, at least in part, by autocrine stimulation from endogenous amphiregulin.

PD098059 significantly inhibited the proliferation of all cell lines suggesting that autocrine activation of EGF receptors leads to activation of the ras/MEK/ERK pathway. This was confirmed by the observation that GST-Elk-1 was significantly phosphorylated by ERK1/ERK2 from CAPAN-2 cells when cultured continuously. Although cell proliferation of CAPAN-2 cells was increased by exogenous EGF, ERK activity was not further increased. This supports the idea that exogenous EGF may activate signalling pathways which are distinct from those activated by endogenous EGF-like factors. Cell cycle analysis shows that the number of CAPAN-2 cells in S phase after 6 hours of continuous culture is reduced in the presence of AG1478 or PD098059. These experiments were performed to show that these inhibitors are also effective in a larger scale study in which larger medium volume and more cells are used.

The kinetics and magnitude of the increase in c-*jun* and c-*fos* mRNA are consistent with the effect of growth factors, such as EGF, on the expression of these genes (Greenberg and Ziff, 1984; Quantin and Breathnach, 1988). The culture environment in which mRNA levels have been monitored in the present investigation permits the interpretation that endogenous growth factors signal through the EGF receptor and ras/raf/MEK pathway leading to an increase in c-*fos* or c-*jun* mRNA. In these experiments, [$^3$H-*methyl*] thymidine incorporation increased with culture time, revealing that the increase in c-*fos* and c-*jun* mRNA precedes that of DNA synthesis and cell proliferation.

It is possible that the observed changes in AP-1 gene expression in CAPAN-2 cells is due to stress arising from the addition of serum-free medium to cells and is not a response to soluble endogenous growth factors. It has been reported that fluid shear-stress can activate the MAPK pathway and induce c-*jun* transcriptional activity in vascular endothelial cells (Li et al, 1996). In the present study, a role for stress, potentially due to the physical addition of serum-free medium, is unlikely since the rate and magnitude of [$^3$H-*methyl*]

© 2001 Cancer Research Campaign

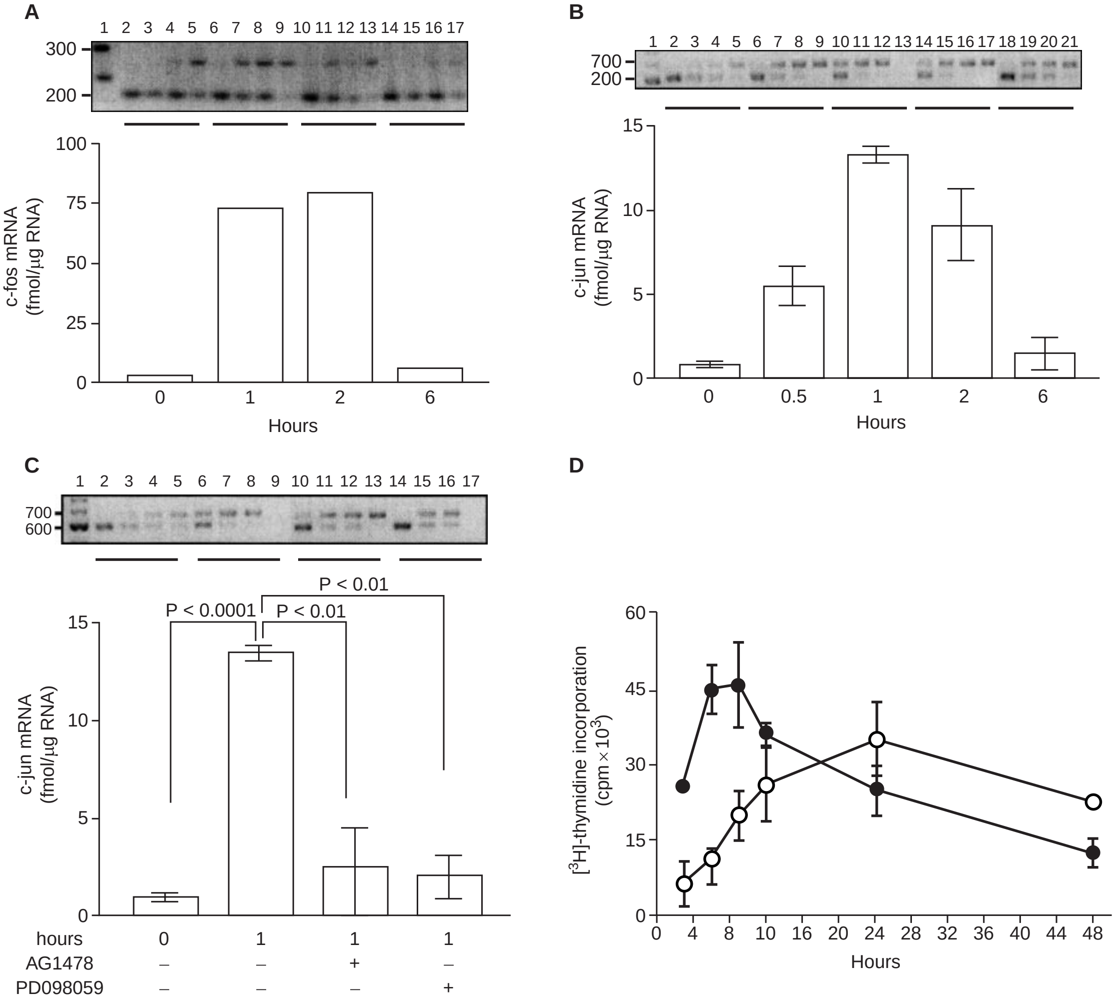

**Figure 7** Quantitative competitive RT-PCR analysis of c-*fos* and c-*jun* mRNA in CAPAN-2 cells. After 0, 0.5, 1, 2 or 6 hours of continuous culture in serum-free medium (**A, B**) or 1 hour of culture in the presence and absence of 10 μM AG1478 or 20 μM PD098059 (**C**), total RNA was extracted from CAPAN-2 cells and mRNA levels were determined as described in the Methods section. The data are expressed as the number of fmoles of c-*fos* or c-*jun* mRNA per μg of total RNA. The amount of cRNA in each RT-PCR tube was 14.1 (lanes 2, 6, 10, 14 and 18), 1.41 (lanes 3, 7, 11, 15 and 19), 0.141 (lanes 4, 8, 12, 16 and 20) and 0.028 fmoles (lanes 5, 9, 13, 17 and 21). Molecular weight standards (base pairs) are shown in lane 1. Data shown represent the mean ± SEM from 2 (**A, B**) or 3 (**C**) separate experiments. [$^3$H-*methyl*] thymidine incorporation into CAPAN-2 cells cultured continuously in 4 ml (solid circles) or 20 ml (open circles) of medium was also determined (**D**)

thymidine incorporation into CAPAN-2 decreased when the volume of serum-free medium was increased. This indicates that the observed growth effects are dependent on the local concentration of endogenous autocrine growth factors in the culture medium and not to shear stress. The result also suggests that the accumulation of endogenous growth factors is slower in 20 ml than in 4 ml of medium. This is consistent with earlier results showing that the proliferation of cells increased with culture time. By establishing a larger ratio of cell number to medium volume, a submaximal proliferative effect was achieved. This experiment also revealed that pancreatic cancer cells can undergo a transient or a sustained proliferative response to endogenous autocrine factors. Sustained mitogenic activity is likely to correspond with the growth phenotype in vivo. Future study of the signalling pathways required for sustained growth may provide new information on the mechanisms responsible for transducing autocrine signals in pancreatic cancer cells.

### ACKNOWLEDGEMENTS

The authors are grateful for the kind gifts of amphiregulin neutralizing monoclonal antibody 16.21.28 from Dr Bruce Cohen, Bristol Myers Squibb (Seattle, WA) and the IGF-I receptor monoclonal antibody αIR3 from Dr Steve Jacobs (Burroughs Wellcome Co., Research Triangle Park, NC). These studies were funded by grants from the Nebraska State Cancer and Smoking Related Disease Program (LB595), the Swedish Cancer Foundation (3450-B95-03xcc and 2870-B96-06xac) and the Carpenter Chair in Biochemistry, Creighton University.

### REFERENCES

Apple SK, Hecht JR, Novak JM, Nieberg RK, Rosenthal DL and Grody WW (1996) Polymerase chain reaction based K-ras mutation detection of

 *© 2001 Cancer Research Campaign*

pancreatic adenocarcinoma in routine cytology smears. *Am J Clin Pathol* **105**: 321–326

Blanco-Aparicio C, Molina MA, Fernandez-Salas E, Frazier ML, Mas JM, Querol E, Aviles FX and de Llorens R (1998) Potato carboxypeptidase inhibitor, a T-knot protein, is an epidermal growth factor antagonist that inhibits tumor cell growth. *J Biol Chem* **273**: 12370–12377

Cook PW, Mattox PA, Keeble WW, Pittelkow MR, Plowman GD, Shoyab M, Adelman JP and Shipley GD (1991) A heparin sulfate-regulated human keratinocyte autocrine factor is similar or identical to amphiregulin. *Mol Cell Biol* **11**: 2547–2557

Ebert M, Yokoyama M, Kobrin MS, Friess H, Lopez ME, Buchler MW, Johnson GR and Korc M (1994) Induction and expression of amphiregulin in human pancreatic cancer. *Cancer Res* **54**: 3959–3962

Funatomi H, Itakura J, Ishiwata T, Pastan I, Thompson SA, Johnson GR and Korc M (1997) Amphiregulin antisense oligonucleotide inhibits the growth of T3M4 human pancreatic cancer cells and sensitizes the cells to EGF receptor-targeted therapy. *Int J Cancer* **72**: 512–517

Greenberg ME and Ziff EB (1984) Stimulation of 3T3 cells induces transcription of the c-*fos* proto-oncogene. *Nature (Lond)* **311**: 433–437

Hahn SA, Schutte M, Shamsul Hoque ATM, Moskaluk CA, da Costa LT, Rozenblum E, Weinstein CL, Fischer A, Yeo CJ, Hruban RH and Kern SE (1996) DPC4, a candidate tumor suppressor gene at human chromosome 18q21.1. *Science* **271**: 350–353

Hollande F, Imdahl A, Mantamadiotis T, Ciccotosto GD, Shulkes A and Baldwin GS (1997) Glycine-extended gastrin acts as an autocrine growth factor in a non-transformed colon cell line. *Gasterenterol* **113**: 1576–1588

Howell GM, Humphrey LE, Ziober BL, Awwad R, Periyasamy B, Koterba A, Li W, Willson JKV, Coleman K, Carboni J, Lynch M and Brattain MG (1998) Regulation of transforming growth factor α expression in a growth factor-independent cell line. *Mol Cell Biol* **18**: 303–313

Jayson GC and Gallagher JT (1997) Heparin oligosaccharides: inhibitors of the biological activity of bFGF on Caco-2 cells. *Br J Cancer* **75**: 9–16

Kobrin MS, Funatomi H, Friess H, Buchler MW, Stathis P and Korc M (1994) Induction and expression of heparin-binding EGF-like growth factor in human pancreatic cancer. *Biochem Biophys Res Comm* **202**: 1705–1709

Ebert M, Yokoyama M, Kobrin MS, Friess H, Lopez ME, Buchler MW, Johnson GR and Korc M (1994) Induction and expression of amphiregulin in human pancreatic cancer. *Cancer Res* **54**: 3959–3962

Korc M (1991) Growth factors and pancreatic cancer. *Int J Pancreatol* **9**: 87–91

Kull FC, Jacobs S, Su Y-F, Svoboda ME, van Wyk JJ and Cuatrecasas P (1983) Monoclonal antibodies to receptors for insulin and somatomedin C. *J Biol Chem* **258**: 6561–6566

Laemmli UK (1970) Cleavage of structural proteins during the assembly of the head of bacteriophage T4. *Nature (Lond)* **227**: 680–685

Landis SH, Murray T, Bolden S and Wingo PA (1999) Cancer Statistics, 1999. *CA Cancer J Clin* **49**: 8–31

Li Y-S, Shyy JY-J, Li S, Lee J, Su B, Karin M and Chien S (1996) The ras-JNK pathway is involved in shear-induced gene expression. *Mol Cell Biol* **16**: 5947–5954

Lynch HT, Smyrk T, Kern SE, Hruban RH, Lightdale CJ, Lemon SJ, Lynch JF, Fusaro LR, Fusaro RM and Ghadirian P (1996) Familial pancreatic cancer: a review. *Semin Oncol* **23**: 251–275

Mulder KM and Brattain MG (1989) Effects of growth stimulatory factors on mitogenicity and c-myc expression in poorly-differentiated and well-differentiated human colon carcinoma cells. *Mol Endocrinol* **3**: 1215–1222.

Munehiro Y, Ebert M and Funatomi H (1994) Amphiregulin is a potent mitogen in human pancreatic cancer cells: correlation with patient survival. *Int J Oncol* **6**: 625–631

Ohmura E, Okada M, Onoda N, Kamiya Y, Murakami H, Tsushima T and Shizume K (1990) Insulin-like growth factors in human pancreatic cancer cell growth. *Cancer Res* **50**: 103–107

Osherov N and Levitzki A (1994) Epidermal-growth-factor-dependent activation of the Src-family kinases. *Eur J Biochem* **225**: 1047–1053

Ötvös FL, Murphy RF and Lovas S (1994) Coupling difficulty following replacement of Tyr with HOTic during synthesis of an analog of an EGF B-loop fragment. *J Pept Res* **53**: 302–307

Qin Y, Ertl T, Cai R-Z, Halmos G and Schally AV (1994) Inhibitory effect of bombesin receptor antagonist RC-3095 on the growth of human pancreatic cancer cells *in vivo* and *in vitro. Cancer Res* **54**: 1035–1041

Qin Y, Ertl T, Cai R-Z, Horvath JE, Groot K and Schally AV (1995) Antagonists of bombesin/gastrin-releasing peptide inhibit growth of SW-1990 human pancreatic adenocarcinoma and production of cyclic AMP. *Int J Cancer* **63**: 257–262

Quantin B and Breathnach R (1988) Epidermal growth factor stimulates transcription of the c-jun proto-oncogene in rat fibroblasts. *Nature (Lond)* **334**: 538–539

Schuger L, Johnson GR, Gilbride K, Plowman GD and Mandel R (1996) Amphireguln in lung branching morphogenesis: Interaction with heparin sulfate proteoglycan modulates cell proliferation. *Development* **122**: 1759–1767

Skullman S, Adrian TE, Permert J, Larsson J (1994) Humoral growth factors in plasma and liver tissue during liver regeneration and malnutrition. *Eur J Gastroenterol Hepatol* **6**: 797–802

Wagner M, Cao T, Lopez ME, Hope C, van Nostrand K, Kobrin MS, Fan HU, Buckler MW and Korc M (1996) Expression of a truncated EGF receptor is associated with inhibition of pancreatic cancer cell growth and enhanced sensitivity to cisplatinum. *Int J Cancer* **68**: 782–787

Wang QJ, Knezetic JA, Schally AV, Pour PM and Adrian TE (1996) Bombesin may stimulate proliferation of human pancreatic cancer cells through an autocrine pathway. *Int J Cancer* **68**: 1–7

© 2001 Cancer Research Campaign
